# Editorial: Cognitive reserve and resilience in aging

**DOI:** 10.3389/fpsyg.2022.1120379

**Published:** 2023-01-05

**Authors:** Renata Kochhann, David Bartrés-Faz, Rochele Paz Fonseca, Yaakov Stern

**Affiliations:** ^1^Research Projects Office, Hospital Moinhos de Vento, Porto Alegre, Brazil; ^2^Department of Medicine, Faculty of Medicine and Health Sciences, University of Barcelona, Barcelona, Spain; ^3^Faculty of Medicine, Federal University of Minas Gerais, Belo Horizonte, Brazil; ^4^Columbia University Vagelos College of Physicians and Surgeons, Columbia University, New York, NY, United States

**Keywords:** cognitive reserve, resilience, brain reserve, brain maintenance, aging

We are delighted to present the nine articles that compose this Research Topic. Consensus operational definitions for concepts of Cognitive Reserve (CR), Brain Maintenance (BM) and Brain Reserve (BR) were recently established by the NIH-funded Reserve and Resilience Collaboratory (https://reserveandresilience.com/). The overall term “resilience” was suggested to encompass all three concepts. The authors adhere to these operational definitions, which increases the clarity and applicability of their findings.

Both CR and BM can be influenced by genetic factors and lifetime exposures. Several papers focused on the nature of these exposures.

Multiple life experiences can be associated with increased CR. Nogueira et al. conducted a systematic review of the most used quantitative measurement methods for CR for in aging. They established that there is no gold standard tool incorporating all proxies and cognitive tests, and highlight the need to develop a more holistic battery for the quantitative assessment of CR.

Rather than reductionistic concepts of simple experiences contributing to reserve, Kempermann proposed a conceptual framework of the “embodied mind in motion”, which recognizes that individual lifestyle is a complex composite of variables relating to both body and mind as well as receiving input and generating output. Hiking, playing musical instruments, dancing and yoga are presented as examples of body-mind activities which be associated with late-life resilience. The article, stresses the concepts of wellbeing and quality of life as drivers of successful interventions, and offers an access point for unraveling the mechanistic complexity of lifestyle-based prevention, including their (neuro-) biological foundations.

Regarding factors that may contribute risk for dementia, Vassilaki et al. present a new tool, the area deprivation index (ADI), which encompass geographic area-based estimates of the socioeconomic disadvantage of neighborhoods, and captures multifactorial contributors to the risk of dementia. They suggest possible mechanisms through which ADI may have an impact on Alzheimer's disease and related dementia outcomes, as well as how resilience can be improved over the lifespan in this context. by considering the ADI as a modifiable risk factor, amenable to policy changes that can affect communities.

Another set of articles investigate CR and BM in the context of their contribution to cognition in the presence of age or disease related brain changes. The studies adhered to the Framework's operation definitions of these concepts. In particular studies of CR incorporated a brain change that results in cognitive change, and a potential moderator of this relationship.

Brichko et al. examine the association of a composite score, composed of years of education, literacy, and vocabulary measures, to the level and rate of change in white matter microstructure, as assessed by diffusion tensor imaging measures. In late middle-aged adults, their composite score was associated with more intact microstructure, consistent with the concept of BM. However, in their older participants, higher composite scores tended to be associated with reduced white matter integrity, suggesting that they contribute to CR by allowing these individuals to maintain cognitive performance in the presence of poorer WM integrity.

Cattaneo et al. investigate a measure of psychological wellbeing, the Sense of Coherence, as potential source of CR. Controlling for brain integrity, as measured by neuro- filament light chain measures, they found that this construct mediated the protective effect of more standard CR proxies on cognitive functions.

Kleineidam et al. explore the potential association of occupational cognitive requirements (OCR) in midlife with BM, brain reserve (BR), or CR. The results support the link between OCR and CR. For example, high OCRS was related to the association between carrying an APOE-ε4 allele and the observed cognitive decline, and it was associated with a later onset but subsequently stronger cognitive decline in individuals converting to dementia.

Böttcher et al. investigate the association and interplay between musical instrument playing during life, multi-domain cognitive abilities and brain morphology in older adults. Participants reporting long-term musical activity during life were compared to controls without musical activity well-matched for reserve proxies of education, intelligence, socioeconomic status and physical activity. Those with musical activity outperformed controls in global cognition, working memory, executive functions, language, and visuospatial abilities, and showed a stronger association between gray matter volumes and cognitive performance than controls.

Jauny et al. synthesize the current state of knowledge from MEG (magnetoencephalography) and EEG (electroencephalography) studies that investigated the contribution of maintenance of neural synchrony and variability of brain dynamics to both cognitive changes associated with healthy aging and the progression of neurodegenerative disease such as Alzheimer disease. They found that both maintenance of young-like synchrony as well as and compensatory adjustments appear to be related with to brain reserve. However, increased synchrony was deleterious in pathological aging.

Habeck et al. evaluated young and old participants during the maintenance phase of a verbal Sternberg fMRI task to identify multivariate activation patterns that increased expression with increased task load. Controlling for structural brain integrity, the load-related increases related *negatively* to mean task accuracy and neuropsychological functioning in the younger group, but positively in the older group. Further, when they prospectively applied the young-derived activation pattern to the older group, the resulting mean load-averaged pattern scores displayed positive correlations with mean task accuracy and neuropsychological functioning. Thus this activation pattern can be considered an implementation of CR.

Overall this set of studies makes a strong contribution to the methodology for studying BM and CR, as well as to potential functional mechanisms that may underly them. We hope that the ideas presented inspire further research in this important area.

## Author contributions

RK wrote the first draft of the editorial. YS, DB-F, and RF edited the editorial. All authors contributed to the editorial revision, read, and approved the submitted version.

